# Seroprevalence and distribution of arboviral infections among rural Kenyan adults: A cross-sectional study

**DOI:** 10.1186/1743-422X-8-371

**Published:** 2011-07-27

**Authors:** Luke E Mease, Rodney L Coldren, Lillian A Musila, Trish Prosser, Fredrick Ogolla, Victor O Ofula, Randal J Schoepp, Cindy A Rossi, Nicholas Adungo

**Affiliations:** 1 Division of Preventive Medicine, Walter Reed Army Institute of Research, 503 Robert Grant Ave., Silver Spring, MD 20910, USA; 2 Department of Preventive Medicine and Biometrics, Uniformed Services University, 4301 Jones Bridge Rd., Bethesda, MD 20814, USA; 3 Department of Emerging Infectious Diseases, United States Army Medical Research Unit-Kenya/Kenya Medical Research Institute, Nairobi, P.O. Box 29893-00202, Kenya; 4 Health Promotion and Wellness, Army Institute of Public Health, U.S. Army Public Health Command, 5158 Blackhawk Road Aberdeen Proving Ground, MD 21010, USA; 5 Centre for Infectious and Parasitic Disease Control Research, Kenya Medical Research Institute, Busia P.O. Box 3-50400, Kenya; 6 Diagnostic Systems Division, U.S. Army Medical Research Institute of Infectious Diseases 1425 Porter St., Fort Detrick, MD 21702, USA

**Keywords:** arbovirus, Kenya, flavivirus, dengue virus, West Nile virus, yellow fever virus, chikungunya virus, Rift Valley fever virus

## Abstract

**Background:**

Arthorpod-borne viruses (arboviruses) cause wide-spread morbidity in sub-Saharan Africa, but little research has documented the burden and distribution of these pathogens.

**Methods:**

Using a population-based, cross-sectional study design, we administered a detailed questionnaire and used ELISA to test the blood of 1,141 healthy Kenyan adults from three districts for the presence of anti-viral Immunoglobulin G (IgG) antibodies to the following viruses: dengue (DENV), West Nile (WNV), yellow fever (YFV), Chikungunya (CHIKV), and Rift Valley fever (RVFV).

**Results:**

Of these, 14.4% were positive for DENV, 9.5% were WNV positive, 9.2% were YFV positive, 34.0% were positive for CHIKV and 0.7% were RVFV positive. In total, 46.6% had antibodies to at least one of these arboviruses.

**Conclusions:**

For all arboviruses, district of residence was strongly associated with seropositivity. Seroprevalence to YFV, DENV and WNV increased with age, while there was no correlation between age and seropositivity for CHIKV, suggesting that much of the seropositivity to CHIKV is due to sporadic epidemics. Paradoxically, literacy was associated with increased seropositivity of CHIKV and DENV.

## Background

**Figure 1:**
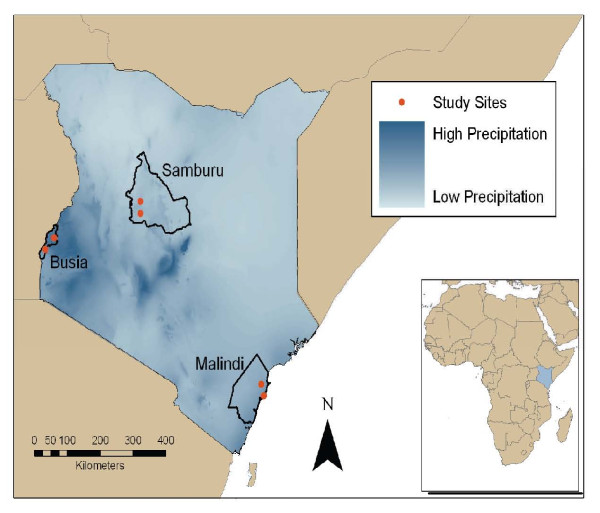
**Map of Kenya with locations of study sites in Busia, Malindi and Samburu districts sampled June-September 2004**. Map is shown in relation to annual precipitation. Darker areas represent greater average precipitation, from a 50 year average. Data from http://www.worldclim.org.

Although there is a significant, increasing worldwide impact of arboviruses from the *Togaviridae*, *Flaviviridae *and *Bunyaviridae *families [1,2], they are poorly understood and controlled. The recent epidemic of Chikungunya virus (CHIKV) in the Indian Ocean Basin[3] has demonstrated the ability of these viruses to spread far beyond traditionally observed areas of distribution[4] and to cause severe morbidity, mortality, and economic harm[5]. Tropical Africa was likely the site of origin of these viruses [6-8] and the burden of disease in this region remains high but much is still not known about their distribution and epidemiology in this region[2]. More is known about these diseases, their vectors and various aspects of their transmission during epidemic periods [3,9-13] than during endemic periods[14]. This lack of epidemiologic knowledge stems in part from a lack of surveillance capacity, with most resources for study and control of these viruses being focused on epidemic periods. Kenya, located in East Africa (Figure 1), is considered to be endemic for arboviruses from the *Togaviridae*, *Flaviviridae *and *Bunyaviridae *families. Competent vectors of these viruses (*Aedes*, *Anopheles *and *Culex *mosquitoes) have been demonstrated throughout Kenya.

Dengue virus (DENV) infection can cause a spectrum of symptoms, from mild, non-specific symptoms to classic dengue fever, with high fevers and severe arthralgia. Reinfection can lead to dengue hemorrhagic fever. In 1982 an outbreak of dengue fever occurred in Kenya[15]. West Nile virus (WNV) infection is generally a self-limited disease with mild symptoms but occasionally causes encephalitis. It has been detected in Kenya's mosquitoes [16]. Yellow fever virus (YFV) can cause severe hepatitis and hemorrhagic fever. In 1992-3 an outbreak occurred in Kenya[17]. YFV, WNV and DENV belong to the *Flaviviridae *family. Infection with CHIKV (*Togaviridae *family) can cause headache, rash, nausea, vomiting and prolonged, debilitating arthralgia. Areas of coastal Kenya were shown to be greatly affected in the recent outbreak of CHIKV [3,18]. Most infections with Rift Valley fever virus (RVFV) (*Bunyaviridae *family) are mild, but a small proportion of infections develop more severe forms of the disease, including ocular, meningoencephalitis or hemorrhagic fever. There have been outbreaks of RVFV in Kenya, most recently in 2006-2007 [13]. Numerous studies have analyzed transmission of this epizootic, arboviral disease during epidemic periods [13,19,20] as well as modeling to predict future outbreaks [21]. There are fewer studies that explore the characteristics of RVF during non-epidemic periods, including several with human data [14,22] and others with animal data [23,24]. Infection with these viruses typically leads to antibody production in the serum. Immunoglobulin M develops acutely and is short-lived, while immunoglobulin G (IgG) develops shortly thereafter and is long-lasting.

In this report, we present the results of a population-based, cross-sectional survey of IgG antibodies against DENV, WNV, YFV, CHIKV and RVFV in Kenyan adults from three districts. The objectives of this study were to determine the endemic prevalence of arboviral illnesses in three ecologically distinct districts in Kenya and to determine the demographic, socioeconomic, and environmental factors associated with previous infection by these viruses.

## Methods

### Ethics statement

The study was approved by Kenya Medical Research Institute's Ethical Review Committee and the Walter Reed Army Institute of Research's Division of Human Subjects Protection. Potential candidates were invited and those meeting all inclusion criteria who were willing to participate and sign a written informed consent were recruited. Study participants were tested for malaria on the same day as the study and, if positive, were treated with an artemisinin-containing combination anti-malarial. This treatment constituted the study's direct benefit to participants.

### Study design

This study utilized a population-based, cross-sectional design. Two villages were selected from each of three districts. The 6 study site villages were selected via two-stage cluster sampling technique, with districts selected *a priori *and sublocation and village subsequently selected randomly, based on population proportional to size. The districts selected, as well as their general geographic location were Malindi (Indian Ocean Coast), Busia (Lake Victoria Basin), and Samburu (semi-arid north). All districts have large rural populations. The districts and villages selected are shown in Figure 1. Following consent, a standardized questionnaire was administered, malaria smear performed and serum sample obtained upon enrollment. Site selection, subject recruitment, questionnaire administration, phlebotomy and ELISA testing occurred between July and Sept 2004.

### Study subjects

The study population consisted of all eligible, consenting adults living in any of the six selected villages. Community members and leaders were engaged prior to recruiting study participants. Meetings were organized wherein the study staff explained the study and answered any questions in an open forum. Upon commencing participant recruitment, enrollment occurred in community centers such as clinics or schools identified by community leaders. Enrollment occurred only after verification of potential subject eligibility, explaining the study in the local language and giving the subjects an opportunity to ask the field staff and study investigators any questions. Criteria for exclusion were: age < 18 years, residence in district <5 years, inability to give informed consent or choosing not to participate. Criteria for inclusion in the study were: absence of any exclusion criteria, demonstration of understanding of the study and expressed willingness to enroll. For all eligible, consenting subjects, witnessed informed consent was obtained.

### Questionnaire

The study questionnaire was developed to obtain demographic, socioeconomic and health information. Local medical authorities participated in design of the questionnaire, which was modeled partly on the Kenya Demographic and Health surveys from 1988-1989[25], 1998[26], and 2003[27]. The questionnaire was piloted in communities similar to those in the actual study and administered by trained interviewers. Before the interview ended, it was verified that recorded questionnaire responses were clear, complete, and internally consistent.

### Laboratory

Using standard precautions, study participants underwent phlebotomy. Blood samples were then transported, under cold storage, to the World Health Organization (WHO) arbovirus reference laboratory in Nairobi, Kenya. There the serum was separated from whole blood, and serum samples were tested for IgG antibodies against the study viruses. Testing was carried out using IgG capture enzyme-linked immunosorbent assay (ELISA). The ELISA reagents used were provided by the Diagnostic Systems Division of the United States Army Medical Research Institute of Infectious Diseases (USAMRIID), USA. The reagents were indicated for research purposes only. Their sensitivity and specificity have not been fully characterized, however, they have been used in numerous serosurveys and epidemics throughout South America, Africa, and Asia

### ELISA antigen

The detergent basic buffer extraction for viral antigen preparation was previously described [28,29]. The viruses utilized for ELISA antigen preparations were Chikungunya (CHIKV), Indo23574 strain; yellow fever (YFV), 17D strain; dengue 2 (DENV), New Guinea C strain; West Nile (WNV), EG 101 strain; Rift Valley fever (RVFV), ZH501 strain. Each virus was propagated in African green monkey kidney (Vero) cells or Vero E6 cells, with the exception of RVFV, which grew in baby hamster kidney (BHK) cells. Briefly, viruses were grown in the appropriate cell line in roller bottles infected at a low multiplicity of infection (MOI) until cytopathic effects (CPE) were significant. Remaining attached cells were scraped into the infected cell suspension and separated from the supernatant by high-speed centrifugation. The pelleted cells were resuspended in borate saline, pelleted again, resuspended in borate saline with detergent, and sonicated. Sonicated cell fragments were pelleted by high-speed centrifugation and the supernatant collected for ELISA antigenic material. The cell lysates of viral antigens were inactivated by addition of 0.3% beta-propiolactone (BPL) and incubation for approximately 72 hr at 4°C, followed by gamma-irradiation. Corresponding negative antigenic material was prepared in the same fashion using uninfected cultured cells. Since RVFV IgG ELISA used an indirect capture sandwich ELISA, the antigenic material was only clarified RVFV infected cell culture supernatant inactivated as described above. All preparations were safety tested to ensure all viruses were inactivated.

Detection of all virus specific IgG antibodies, except RVFV, utilized the indirect ELISA format previously described [28,29]. Briefly, one half of a 96-well polyvinylchloride (PVC) microtiter plate was coated directly with 100 µl/well of inactivated, detergent-extracted virus infected cell lysate in 0.01 M phosphate-buffered saline (pH 7.4) with 0.01% thimerosal (coating buffer) at a predetermined optimal dilution. Antigen was optimized using known positive human or animal serum. The other plate half was coated with 100 µl/well of the appropriate corresponding negative cell antigenic preparation. Plates were wrapped in plastic wrap and incubated at 4°C overnight.

The next day plates were washed three times with wash buffer (coating buffer and 0.3% Tween-20). All subsequent reagents added to the plates were diluted in reagent buffer (wash buffer containing 5% skim milk). After the addition of each reagent, the plates were incubated in a moist environment at 37°C for 1 hr and then washed three times. Serum samples were diluted to obtain a final 1:100 dilution when added into both positive and negative coated wells. Positive control serum from native infections or vaccine recipients was used to monitor reproducibility of assays. Four negative control sera were included for each assay to determine the optical density (OD) cut-off value. After addition of serum, plates were incubated for 1 hr at 37°C and subsequently washed. Conjugate was a mouse anti-human IgG (FC) serum conjugated with peroxidase (Accurate Chemical & Scientific Corp, Cat. No. JMH035103, Westbury, NY) at a dilution determined by previous titration. The substrate, 100 µl of 1:1 ABTS (2.2-azino-di [3-etil-benztiazolin sulfonato] (Kirkergard and Perry, Cat. No. N8 50-62-00, Gaithersburg, MD) and hydrogen peroxide (H_2_O_2_), was added to each well to complete the reaction. After 30 min incubation, plates were read spectrophotometrically at 405 nm. For detection of RVFV antibodies, a capture or sandwich assay was used. This assay was performed as described above, except the PVC microtiter wells were initially coated with an optimized dilution of a nucleocapsid-specific monoclonal antibody that captures the inactivated RVFV antigen.

The adjusted OD was calculated by subtracting the OD of the negative antigen coated wells from the positive antigen coated wells. The OD cut-off was calculated as the mean of the adjusted OD of the negative control sera plus three times the standard deviations, generally an OD of ≤ 0.2 at a 1:100 sample dilution. A serum sample was considered positive if the adjusted OD value was greater than or equal to the assay cut-off or 0.2, which ever value was higher.

### Data analysis

The main outcomes of interest were IgG antibodies against: DENV, WNV, YFV, flaviviruses (using combined data from YFV, DENV and WNV), CHIKV and RVFV. For each outcome we calculated overall seropositivity rates. The following variables from the questionnaire were selected, *a priori*, for analysis: age (18-25, 26-35, 36-45 and 46+ years old), sex, literacy (defined by self-reported ability to read a newspaper), occupational group (agriculture, home duties or other) and roofing type (thatch, corrugated metal or wood/mud/concrete). Not all variables from the questionnaire were included in the data analysis. Selection of variables to include was based on author consensus and pertinence to arbovirus epidemiology (i.e. many of the questions regarding malaria-specific risk factors were omitted).

IgG status was recorded and analyzed as a dichotomous variable (positive or negative). Data entry described previously[30]. Stata version 10 IC for Window (Stata Corp., College Station, TX) was used for all statistical analysis.

Prior to commencement of the study, sample size calculations determined that at least 204 participants would be required from each district, using a hypothetical comparison ratio positive (percentage IgG positive) of 0.00, a hypothesized ratio positive (level considered epidemiologically significant) 0.05, alpha 0.05, power 0.9 and one-sided testing.

We calculated descriptive statistics for questionnaire variables. χ^2 ^and Fisher's exact tests were used to evaluate heterogeneity of rates among the different districts. Using unadjusted logistic regression models we examined the effects of sex and age, individually, on the odds of seropositivity to alphaviruses, flaviviruses, YFV, DENV and WNV. We subsequently evaluated self-reported literacy, home roofing material, and occupation on odds of seropositivity, using logistic regression models adjusting for age and sex.

Increased mobility (measured by bicycle ownership), having children at home and differences in housing type (measured by roof type) were considered as possible confounders of the effect of literacy on seropositivity. Separate logistic models were constructed, controlling for age, sex and, one by one, these possible confounders. District of residence was considered a possible confounder of the effect of roofing material; to control for this the effect of roofing material was evaluated by logistic regression modeling after stratifying by district of residence. For RVFV, associations between seropositivity and age, sex, formal education, home roofing material and occupation were examined using Fisher's exact test. To further increase statistical power, these analyses were performed twice using 2-way Fisher's exact test, excluding either Malindi or Samburu district. If data was missing regarding the outcome or covariate of interest in statistical analysis, this observation was omitted.

## Results

Demographics of the study population are summarized in Table 1. The sampled areas represent a predominantly rural populace. 1,141 total subjects were enrolled, representing 27.8% of the adult population of the selected villages. 461 participants were from Busia, 442 from Malindi and 238 were from Samburu. 731 (64.1%) were female and 410 (35.9%) were male. There was a difference in sex distribution among the different age ranges in all districts, with younger females more proportionally represented than younger males (p < 0.001). There was, however, no statistically significant difference in the age or sex distribution of subjects among the three districts. Results of serological testing, stratified by district, are summarized in Table 2. Previously published data on malaria parasitemia[30], representing the same participants and collection dates as this study, are included in the table for comparison purposes.

**Table 1:** Sociodemographic characteristics of study participants by district (n = 1141)

District	Busia n = 461	Malindi n = 442	Samburu n = 238	Missing	Total n = 1141
**Sex**				2	
Female	298	281	152		731
Male	161	161	86		408

**Age**				0	
18-25	98	116	52		266
26-35	109	121	56		286
36-45	90	85	44		219
> 45	164	120	86		370

**Literacy^a^**				8	
Easily	183	203	40		426
With difficulty	140	53	27		220
Not at all	136	184	167		487

**Occupation**				31	
Other^b^	128	159	50		337
Agriculture	300	223	83		606
Home Duty	13	50	104		167

**Roof Type**				0	
Wood, Mud, Concrete	2	3	108		113
Corrugated Metal	187	108	42		337
Thatch	272	331	88		691

**Table 2:** Prevalence of anti-arboviral IgG antibodies by district, Kenya, September 2004 (n = 1141)

	Busia % (n)^c^	Malindi	Samburu	Missing	Total Positive
**Any Arbovirus**	62.09 (285)	51.58 (228)	6.47 (15)	8	46.60 (528)
**Flaviviruses (all pos.)^d,e^**	6.97 (32)	37.78 (167)	4.31 (10)	8	18.50 (209)
**Flaviviruses (multiple pos.)**	1.96 (9)	20.81 (92)	0.86 (2)	14	9.09 (103)
**DENV (all pos.)**	1.96 (9)	34.17 (149)	1.72 (4)	14	14.40 (162)
**DENV (sole pos.)^f^**	0.44 (2)	13.67 (60)	1.29 (3)	14	5.77 (65)
**WNV (all pos.)**	4.36 (20)	18.81 (82)	2.16 (5)	14	9.49 (107)
**WNV (sole pos.)**	3.05 (14)	2.29 (10)	1.29 (3)	14	2.40 (27)
**YFV (all pos.)**	3.49 (16)	19.27 (84)	1.72 (4)	14	9.23 (104)
**YFV (sole pos.)**	1.53 (7)	1.15 (5)	0.86 (2)	14	1.24 (14)
**CHIKV**	59.91 (275)	24.77 (108)	0.00 (0)	14	34.00 (383)
**RVFV**	0.00	0.23 (1)	3.02 (7)	32	0.72 (8)
**Malaria^g^**	22.34 (103)	7.24 (32)	1.68 (4)	0	12.2 (139)

Table 3 summarizes the association between sociodemographic factors and anti-arboviral IgG seropositivity using logistic regression. There was significant heterogeneity of seroprevalence for all arboviruses by district, using χ^2 ^and Fisher's exact test with p < 0.001 in all cases, but no significant heterogeneity of seroprevalence between study sites within the same district. For flaviviruses there was no significant difference in the proportion of seropositive individuals between Busia and Samburu; in both districts seroprevalence was <5% for all individual flaviviruses. All RVFV positives were from the Samburu district except one. This individual was from Malindi district and reported never having lived outside that district.

**Table 3:** Factors associated with anti-arboviral IgG seropositivity among rural adults in logistic regression, Kenya, September 2004

	Flavivirus	DENV	WNV	YFV	CHIKV
**District**	OR (95% CI)^h^	OR (95% CI)	OR (95% CI)	OR (95% CI)	OR (95% CI)
Busia	Reference	Reference	Reference	Reference	Reference
Malindi	**8.1 (5.39-12.18)^i^**	**25.96 (13.03-51.69)**	**5.08 (3.06-8.45)**	**6.61 (3.8-11.48)**	**0.22 (0.17-0.29)**
Samburu	0.6 (0.29-1.25)	0.88 (0.27-2.88)	0.48 (0.18-1.31)	0.49 (0.16-1.47)	No values

**Sex**					
Female	Reference	Reference	Reference	Reference	Reference
Male	1.08 (0.79-1.48)	1.07 (0.76-1.52)	1.41 (0.94-2.11)	1.31 (0.87-1.97)	1.04 (0.81-1.35)

**Age**					
18-25	Reference	Reference	Reference	Reference	Reference
26-35	1.54 (0.94-2.5)	**1.51 (0.86-2.65)**	**1.8 (0.89-3.61)**	**2.28 (1.02-5.07)**	1.16 (0.81-1.65)
36-45	**2.24 (1.37-3.65)**	**2.77 (1.6-4.79)**	**2.6 (1.3-5.2)**	**4.5 (2.09-9.71)**	0.89 (0.6-1.31)
46+	**2.09 (1.33-3.27)**	**2.24 (1.34-3.75)**	**2.64 (1.39-5)**	**3.87 (1.85-8.07)**	1.2 (0.86-1.68)

**Literacy^j,a^**					
Easily	Reference	Reference	Reference	Reference	Reference
With difficulty	0.7 (0.48-1.04)	**0.59 (0.36-0.99)^h^**	0.57 (0.3-1.08)	0.58 (0.31-1.09)	0.96 (0.68-1.35)
Not at all	0.9 (0.62-1.31)	0.74 (0.49-1.12)	0.97 (0.59-1.58)	0.76 (0.46-1.26)	**0.45 (0.33-0.62)**

**Occupation^j^**					
Other^b^	Reference	Reference	Reference	Reference	Reference
Agriculture	0.84 (0.59-1.2)	0.82 (0.55-1.22)	1.11 (0.69-1.78)	0.97 (0.6-1.56)	1.22 (0.91-1.64)
Home Duty	0.62 (0.36-1.06)	0.67 (0.37-1.22)	0.55 (0.24-1.26)	0.62 (0.28-1.37)	**0.23 (0.14-0.4)**

**Roof Type^j^**					
Wood, Mud, Concrete	Reference	Reference	Reference	Reference	Reference
Corrugated Metal	**6.5 (1.98-21.31)**	**7.62 (1.81-32.13)**	**10.69 (1.44-79.54)**	**4.92 (1.15-21.12)**	**76.86 (10.6-557.45)**
Thatch	**11.86 (3.7-38.03)**	**13.15 (3.19-54.2)**	**15.67 (2.15-114.22)**	**7.8 (1.88-32.41)**	**65.13 (9.03-469.88)**

Lower seroprevalence of CHIKV antibodies was noted among illiterate participants when compared to those who reported being able to read easily (OR 0.45 (0.33-0.62)). Similarly, for DENV, those who reported being able to read with difficulty had lower odds of seropositivity than those would could read easily (OR 0.59 (0.36-0.99)). Having children at home, increased mobility (measured by bicycle ownership) and housing type (measured by roofing type) were considered as possible confounders in this association but the statistically significant association of decreased seroprevalence among illiterate study participants persisted after controlling for each of these variables individually.

For CHIKV, higher odds of antibody seroprevalence were seen among those with corrugated metal and thatch roofs and as compared to those with wood/mud/concrete roofing. Corrugated metal and thatch roofing were associated with increased odds of antibodies against flaviviruses. Similarly, significantly increased odds of seropositivity to DENV, WNV, and YFV were noted among those with corrugated metal or thatch roofs. To control for potential confounding due to use of different types of roofing materials in distinct geographic settings, logistic regression was performed after stratifying by district. No significant association remained for any virus or group following stratification, which suggests that associations between housing materials and arbovirus seropositivity are strongly confounded by district of residence.

Possible associations between RVFV antibody seropositivity and district of residence, age, sex, literacy, occupation, or roofing material were tested using Fisher's exact test. RVFV antibody seropositivity was significantly associated with district of residence (p < 0.001). No statistically significant association was noted for age, sex, literacy, occupation, or roofing material, both within analyses that included all districts, and those that excluded Malindi and Busia in turn.

## Discussion

Arboviral infections are considered a serious public health problem in tropical Africa; this study confirms that a large proportion of adults in rural Kenya have been infected by arboviruses at some point in their life. The true burden of disease from arboviral infections cannot be measured with a cross-sectional study, but this data suggests that it is high in rural Kenya. Furthermore, this study only detected those who survived arboviral infection, ignoring the burden caused by mortality from arboviral infections in the communities sampled.

The proportion of individuals with evidence of arboviral infection differed significantly between districts; however no difference was detected between different study sites within the districts tested. A different virus was found to be most prominent in each of the three districts tested; CHIKV infection was most prevalent in Busia, flavivirus infection in Malindi and RVFV in Samburu. This finding suggest that the risk of infection from arboviral disease differs significantly between different areas of Kenya, but that within a district, the risk is relatively homogenous. This has public health implications, suggesting that prevention programs (or in the case of an outbreak, investigation and intervention efforts) should be virus or genus-specific. Furthermore, these efforts might be most effectively targeted at a district level, as opposed to country-wide, which would be too broad or village-specific, which might be too narrow.

The absence of association between age and CHIKV seropositivity suggests that the predominant cause of seropositivity among this group of Kenyan adults may be exposure to infected mosquitoes during one or more outbreaks. In contrast, for flaviviruses, the association of increasing age with increasing seroprevalence suggests more stable rates of ongoing transmission among the population tested.

The finding that illiteracy seems to be protective against infection with CHIKV and DENV was not entirely unexpected, as a similar finding of lower odds of malaria parasitemia among illiterate participants was noted in a previous publication from this study [30]. It is still surprising, as literacy is generally associated with increased socioeconomic status, increased access to preventive measures and more developed habitation, all which would presumably lead to a lower risk of exposure to infected mosquitoes. Possible explanations for this finding include increased mobility among those who are literate and school attendance by their children, allowing more potential for exposure at multiple locations, among the tested adults and their children. However, the association of increased seropositivity among literate participants persisted even after controlling, individually, for increased mobility (measured by bicycle ownership) and having children at home. Different housing type (as measured by roofing type) was also considered as a possible confounder but the association of increased seropositivity among literate participants also persisted after controlling for this. This paradoxical finding may be due to bias or confounding from an unmeasured variable, but it appears to be internally valid, since it is consistent for multiple agents (CHIKV, DENV and malaria.)

Housing type (measured by roofing type) may be an important risk factor in exposure to infected mosquitoes. However, the apparent association between roofing type and arbovirus seroprevalence lost statistical significance after controlling for district of residence, suggesting that any association between housing materials and arbovirus seropositivity is strongly confounded by district of residence.

This study is limited by the lack of validated ELISA assays and plaque reduction neutralization tests (PRNTs) to increase the specificity of ELISA testing and reduce potential cross reactivity at the time the study was conducted. The lack of non-validated assays engenders inherent uncertainty in the results of this study. Further uncertainty is engendered by the unavailability of confirmatory plaque reduction neutralization tests (PRNTs). This highlights the need for the increased availability of affordable, validated assays for use in clinical, surveillance and research settings. An important limitation is potential cross-reactivity between CHIKV and O'nyong-nyong virus (ONNV). Belonging to the same genus and causing similar symptoms, CHIKV and ONNV are also highly related. Because of similar antigenic structure, there is often a high level of antibody cross-reactivity. Cross-reactivity between these viruses is well documented [31,32] and so high that any positive CHIKV result could be almost equally due to either CHIKV or ONNV. The results of this study should be interpreted in light of this. Although not at the level expected for CHIKV and ONNV, cross-reactivity between the flaviviruses tested in this study is also well-documented and the results should be interpreted accordingly. Another limitation is that the data were collected several years ago, notably prior to the most recent outbreaks of CHIKV and RVFV affecting Kenya. While these data may not reflect the current status of seroprevalence in the areas tested, they also provide possible pre-outbreak baselines of CHIKV seroprevalence in coastal Kenya and RVFV seropositivity in central Kenya. The exclusion of individuals under the age of 18 is another potential limitation, as the pediatric population in these communities must play an important role in the epidemiology of the infections studied.

## Conclusions

In conclusion, we found that a large proportion of rural Kenyan adults have been infected by arboviruses. Several sociodemographic factors were associated with arboviral seropositivity. Most of these were expected, for example the difference in seroprevalence seen between districts and the increasing odds of seropositivity seen with increasing age for flaviviruses. Other findings, such as the absence of association between CHIKV and age, and the apparent protective effect against arboviral infection among illiterate participants have not been well described in the literature and merit further study.

## Abbreviations

CHIKV: Chikungunya virus; DENV: dengue virus; ELISA: enzyme-linked immunosorbent assay; OONV: O'nyon-nyong virus; PRNT: plaque reduction neutralization test; RVFV: Rift Valley fever virus; WNV: West Nile virus; YFV: yellow fever virus.

## Competing interests

The authors declare that they have no competing interests.

## Authors' contributions

LM served as Principal Investigator on the study, performed all data analysis and oversaw manuscript preparation, protocol design and execution. RLC was involved in all aspects of protocol design and execution, data analysis, and manuscript preparation. LAM assisted in manuscript preparation, protocol design and execution. TP and FO designed the sociodemographic questionnaire, supervised field workers, and assisted in manuscript preparation. VOO oversaw laboratory testing and assisted in manuscript preparation. RJS and CAR assisted in assay and laboratory preparation, trained laboratory staff, served as advisors during the testing and assisted with manuscript preparation. NA conceived the study, supervised field and data entry personnel, and assisted with manuscript preparation. All authors read and approved the final manuscript.

## Endnotes

a. Literacy was measured by self-reported ability to read a newspaper.

b. Other category for Occupation includes those who reported work in small business, large business, civil service, transport, tourism, formal employment, work as casual laborers or unemployed.

c. % and (n) represent the percent and number within the given district with antibodies to the corresponding virus.

d. Pos., positive.

e. Flavivirus category presents summation for specific tests for DENV, WNV and YFV.

f. Sole pos. indicates number of individuals positive for only that flavivirus and no other flavivirus. Those included may or may not be positive for alphavirus and RVF.

g. Malaria data included for comparison purposes only. It represents same participants and collection dates as this study, previously published[30].

h. OR, odds ratio; CI, confidence interval.

i. Statistically significant odds ratios (P-value ≤ 0.05) are indicated in bold.

j. Controlled for age and sex in multivariate logistic regression.
